# Uncovering Dislocation- and Precipitate-Induced Viscoplastic Damage in Al-Zn-Mg Alloy

**DOI:** 10.3390/ma16103769

**Published:** 2023-05-16

**Authors:** Yunlong Zheng, Ning Guo, Bingtao Tang, Baoyi Su, Qingjun Zhou

**Affiliations:** 1 School of Mechanical Engineering, Qilu University of Technology (Shandong Academy of Sciences), Jinan 250353, China; zyl19862127828@163.com (Y.Z.); tbtsh@hotmail.com (B.T.); subaoyi10086@gmail.com (B.S.); 2 Shandong Institute of Mechanical Design and Research, Jinan 250031, China; 3 Capital Aerospace Machinery Co., Ltd., Beijing 100076, China; ningguo@qlu.edu.cn

**Keywords:** Al-Zn-Mg alloy, viscoplastic constitutive model, precipitates, damage, dislocations

## Abstract

The existing phenomenological theories of plastic forming of sheet metal lack the predictability of the influences of dislocations and precipitates on viscoplastic damage in Al-Zn-Mg alloys. This study examines the evolution of grain size that occurs when the Al-Zn-Mg alloy undergoes a hot deformation process, specifically concentrating on dynamic recrystallization (DRX). The uniaxial tensile tests are conducted at deformation temperatures ranging from 350 to 450 °C and strain rates of 0.01–1 s^−1^. The intragranular and intergranular dislocation configurations and their interactions with dynamic precipitates are revealed by transmission electron microscopy (TEM). In addition, the MgZn_2_ phase induces microvoid formation. Subsequently, an improved multiscale viscoplastic constitutive model is established that emphasizes the effect of precipitates and dislocations on the evolution of microvoid-based damage. Using a calibrated and validated micromechanical model, the simulation of hot-formed U-shaped parts is conducted through finite element (FE) analysis. During the hot U-forming process, the formation of defects is expected to have an impact on both the distribution of thickness and the level of damage. In particular, the damage accumulation rate is influenced by temperature and strain rate, and local thinning is caused by the damage evolution of U-shaped parts.

## 1. Introduction

Environmental protection and energy efficiency are common concerns of the automotive industry, in which crashworthiness and light weight are the ultimate targets of automotive body design and manufacturing. Due to their high strength, corrosion resistance, and low density, the 7xxx series aluminum alloys (Al-Zn-Mg) have found widespread use in the production of car bodies [1]. Formability defects and performance control issues in Al-Zn-Mg alloys during hot stamping [2] impede the production of curved and unique-shaped thin-wall parts essential to the manufacture of energy-efficient automobile bodies [3]. In particular, the microstructural evolution [4] and complex stress states [5] that occur during hot stamping, especially during industrial processes with varying temperature values and transient stamping rates [6,7], can result in mixed and complex effects, including grain size evolution and microvoid damage [8].

The efficiency of Al-Zn-Mg alloys during forming processes is closely linked to their constituent components and mechanical properties [9,10,11]. The abundance and strong pinning effect [12,13] of the metastable η precipitate [14,15], which is a well-known constituent strengthening factor, make it the primary contributor to the material strengthening in the peak-aged (T6 and T651) AA7075 alloy by affecting the dislocation distribution. Zhou et al. [16] demonstrated that the dynamic strain aging effect induced by the dislocation pinning delay fracture; simultaneously, the precipitates could significantly improve the strength and toughness at higher solution temperature values and lower strain rates. Murchu et al. [17] noted that the major obstacles to dislocation motion are nanoscale precipitates, which result in both hardening and fracture. In addition, Ying et al. [18] found that the hot plastic deformation induces damage evolution in the AA7075 alloy through void nucleation, growth, and coalescence, which are significantly influenced by the dislocation density and grain size. However, the interactions between precipitates and dislocations could actually lead to damage and finally fracture during the hot plastic deformation process of aluminum alloys. Importantly, Han et al. [19] identified the presence of intergranular precipitates and the combination of free precipitation zones as the primary fracture mechanism. Dixit et al. [20] indicated that dislocations shear or bypass precipitated particles according to their size. Consequently, further experimental verification for the combined effects of dislocation configurations and precipitate-induced damage is required.

In recent years, for AA7075 alloy during the hot-stamping process, researchers have developed and proposed various physics-based constitutive models and phenomenological constitutive models to predict damage evolution. Saravanan et al. [21] noted that the phenomenological Arrhenius model can well capture the flow stress of extruded Al-Mg-Zn-Cu alloys. It was noted that the Johnson–Cook model [22], the Fields–Backofen model, and the Arrhenius model [23] can also accurately describe the flow stress pattern at the macroscopic level. However, the microstructural evolution cannot be monitored and revealed during hot plastic deformation [24], and therefore, it is not possible to predict both damage and fracture events. The viscoplastic constitutive model considers internal state variables, such as dislocation density, grain size, and damage to capture microstructure changes. Compared with traditional phenomenological models, this model has received widespread attention [25]. In predicting macroscopic flow stress, Lu et al. [26] developed a viscoplastic model that utilized internal variables to capture the flow stress behavior of AA7075 alloy at elevated temperatures. By performing a range of uniaxial tensile tests under isothermal conditions, Rong et al. [27] developed an improved model for continuous damage to describe the thermomechanical response and damage progression of the AA7075 alloy within a temperature range of 300–400 °C. Chen et al. [28] presented a noteworthy study in which they introduced a group of plane stress models based on dislocation mechanics to simulate the deformation behavior of aluminum alloys during plastic forming. The models were utilized to anticipate flow behavior at different strain rates within the temperature range of 300–400 °C. Li et al. [29] developed a viscoplastic model that takes into account the effects of precipitates, solute concentration, and dislocation evolution. On top of that, Xiao et al. [30] devised a series of viscoplastic models based on grain size, dislocation density, and damage. The authors accurately anticipated the flow stress and microstructural evolution of AA7075 alloy. Tang et al. [31] established a viscoplastic constitutive model based on the damage mechanism by studying the hot deformation behavior and microstructure evolution of AA7075 aluminum alloy. The calculated results were compared with experimental results, and the comparison showed that the established mathematical model can effectively predict deformation at high temperatures. However, the combined impact of dislocation configurations and precipitate-induced microvoids on the damage evolution is not quite clear. In addition, the existing viscoplastic constitutive models lack the ability to accurately predict the relationship between dislocations and formability defects.

Under this direction, in this study, uniaxial tensile tests of the AA7075 alloy sheet at elevated temperature values were carried out. The influence of the interactions between precipitates and dislocations on the evolution of microvoid-based damage was clarified by conducting TEM, electron backscatter diffraction (EBSD), and scanning electron microscopy (SEM) measurements. On this basis, an improved multiscale viscoplastic model was established by comprehensively considering grain size, dislocation density, DRX, and precipitate-induced damage. Then, hot tensile experiments were conducted on the notched AA7075 specimen, and a FE simulation was performed to validate the viability of the proposed model. Ultimately, to predict the damage distribution at both the mesoscopic and macroscopic levels, simulations were performed on the hot-formed U-shaped components. Our work provides valuable theoretical insights and practical guidance for further improving the formability of complicated thin-walled parts made of Al-Zn-Mg alloys in mass production.

## 2. Experimental Procedures

To uncover how the interplay between dislocations and precipitates in AA7075 alloy influences the damage evolution during deformation, experiments were conducted at both macroscopic and mesoscopic levels. The experiments included notched specimen microstructure characterization as well as hot tensile and U-shaped part hot-forming tests.

### 2.1. Uniaxial Hot Tensile Tests

In this study, an Al-Zn-Mg alloy sheet with the designation AA7075-T6 and a thickness of 1.5 mm was utilized, and Table 1 provides the composition (in weight percent) of the selected case-study alloy.

**Figure 1:**
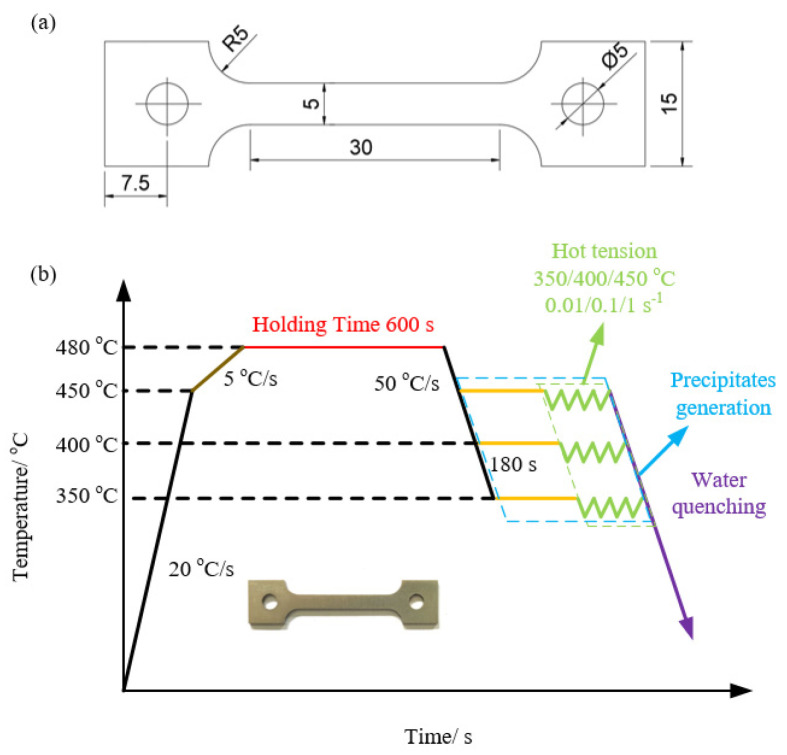
(**a**) Schematic diagram of the tensile specimens (mm); (**b**) schematics of the process routine for the execution of the hot tensile tests.

A series of tensile tests at elevated temperatures were performed using a Gleeble 3500C testing system. The dimensions of the samples are shown in Figure 1a. The designated procedure of tensile tests at elevated temperatures according to the actual requirements for aluminum alloys in the industry is depicted in Figure 1b. At the initial heating stage, dog-bone specimens were heated to the temperature of 480 °C at initial heating rates of 20 °C/s and subsequent rate of 5 °C/s. Then, the samples were held for 600 s and treated with a solid solution to ensure microstructure homogenization. It should be observed that the so-called precipitate generation stage, as depicted in Figure 1b, occurs during the holding and stretching process at various temperature values. The specimens were subjected to deformation temperatures of 350 °C, 400 °C, and 450 °C, followed by fracture testing at strain rates of 0.01 s^−1^, 0.1 s^−1^, and 1 s^−1^, respectively. Afterward, water quenching was used to preserve the morphological characteristics of the specimens deformed under high-temperature conditions.

### 2.2. Microstructure Characterization

To carry out EBSD measurements, the samples were first mechanically ground with 1000 # SiC grinding paper and then mechanically polished with 1.5 μm adamantine plaster. In order to achieve high-quality EBSD patterns, the specimens underwent electropolishing using a solution consisting of 50 mL perchloric acid (5%) and 950 mL ethanol (95%). The EBSD maps were recorded using JSM-7900F equipment with a 20 kV beam voltage, step size of 1 μm, and 200× magnification. The scanning was carried out at least 300 μm away from the sample boundary to prevent any drifting effects near the boundary. A directional imaging microscope was used to analyze the EBSD images. The definition and recognition of the grains were based on setting the minimum confidence index to 0.1 and the grain tolerance angle to 15°.

To thoroughly investigate the morphological characteristics and the combined effects of the dislocation configurations and precipitates, TEM analysis of the intragranular and intergranular dislocation configurations, as well as calibration of the precipitates, was carried out. The detailed sample preparation process for the TEM observation was as follows. Specimens with 3 mm in thickness underwent mechanical grinding until a thickness of 50 μm was achieved. Then, an MTP-1 dual-jet electrolyzer was employed for thinning. It should be noted that the dual-jet thinning parameters were as follows: φ (HNO_3_):φ (CH3OH) = 1:3, thinning voltage: 20 mV, current: 40–50 mA, and temperature: −30–−40 °C. Subsequently, the thinned specimens were analyzed by TEM imaging to characterize the dislocation configurations and types of precipitates.

### 2.3. Hot Stretching of Notched Specimens

**Figure 2:**
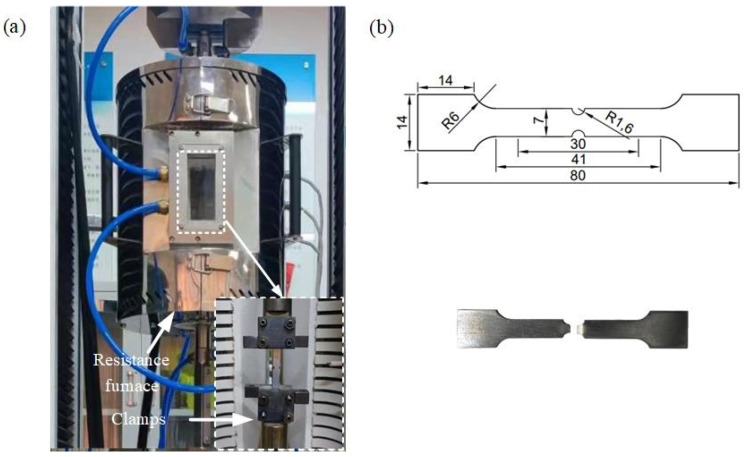
Hot stretching of notched specimens: (**a**) electronic universal testing machine and resistance furnace; (**b**) geometrical dimensions of the specimen (mm) and fractured notched specimen.

The fracture strain of the AA7075 alloy in the hot stretching process was studied using customized notched specimens. The hot stretching experiment was conducted at a loading speed of 1.5 mm/s and a temperature range of 350 and 400 °C, with an average strain rate of approximately 0.001 s^−1^. The strain before fracture of the sample at 350 °C and 400 °C was 0.804 and 0.681, respectively. The hot stretching equipment consisted of a WDW-200E electronic universal testing machine and an HSGW-1200A resistance furnace, as displayed in Figure 2a. Moreover, the resistance furnace provided a heated and stable insulated environment. The geometrical sizes of the notched samples and the fractured samples are shown in Figure 2b.

### 2.4. Hot-Stamped U-Shaped Parts

**Figure 3:**
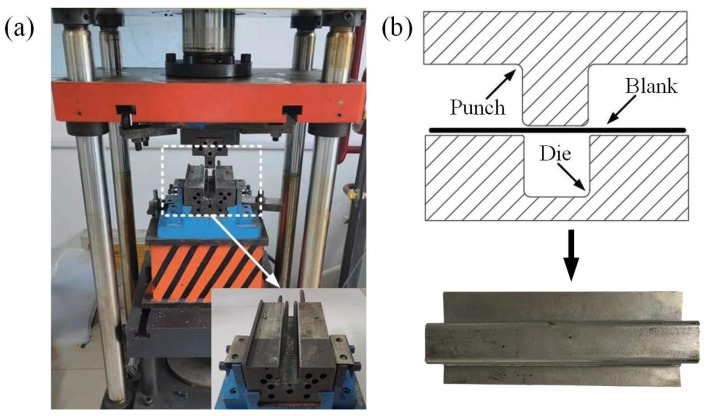
(**a**) U-forming platform; (**b**) schematic diagram and U-shaped parts.

To investigate the formability of the AA7075 sheet during the U-shaped component-shaping process, a platform was employed. The experiments were conducted at a punch speed of 20 mm/s and within the designated temperature range of 350–450 °C. The U-forming device, illustrated in Figure 3a, consisted of a U-shaped punch and die. Furthermore, the hydraulic press YZ32-40, capable of applying a maximum downward force of 400 KN, was used to provide the forming load. The schematic diagram and the stamped U-shaped part are shown in Figure 3b.

## 3. DRX Characterization and Precipitate Evolution

The evolution of DRX and precipitates has a non-negligible effect on the forming process of the AA7075 alloy during hot formation. The microstructure of the specimen was characterized in this section. The grain orientation, grain size, and DRX were characterized by EBSD near the fracture area. The combined effects of dislocation configuration and precipitates were further elucidated by conducting the TEM analysis. The DRX and precipitate-induced damage evolution of the AA7075 alloy under hot deformation was also systematically analyzed.

### 3.1. Grain Morphology and DRX Evolution

**Figure 4:**
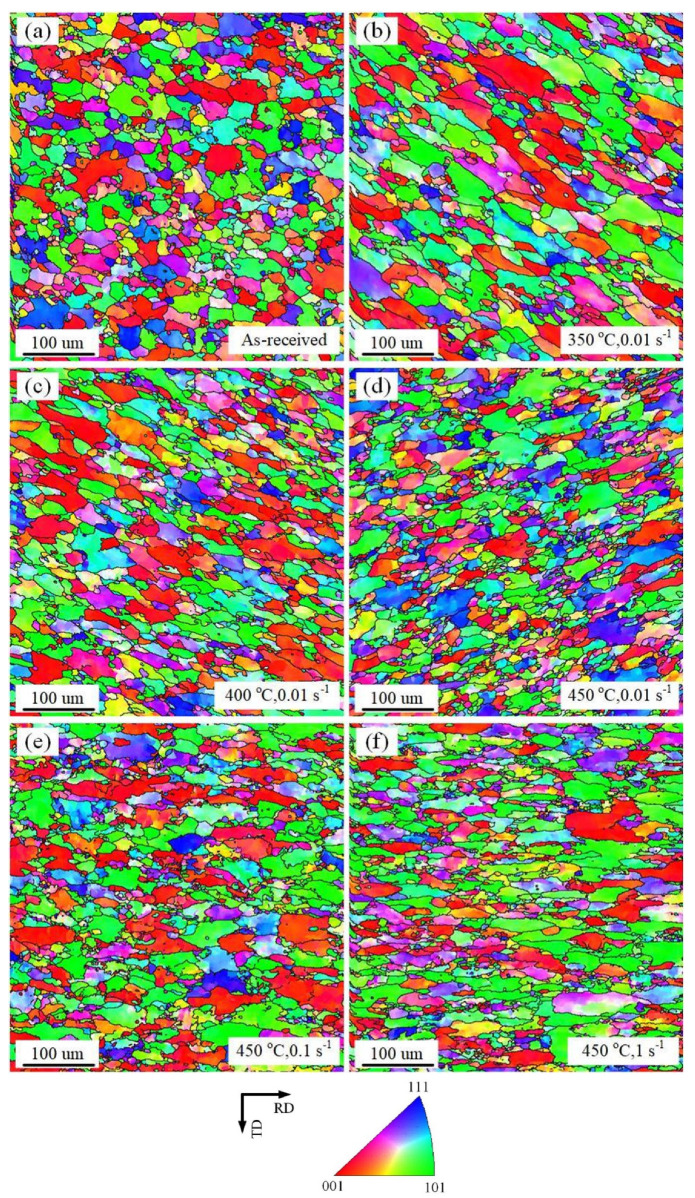
IPF images (**a**) of the as-received samples and deformed ones with (**b**) 350 °C, 0.01 s^−1^ (**c**) 400 °C, 0.01 s^−1^ (**d**) 450 °C, 0.01 s^−1^ (**e**) 450 °C, 0.1 s^−1^, and (**f**) 450 °C, 1 s^−1^.

Grain size and crystal orientations are considered the most significant factors influencing the DRX evolution of AA7075 alloy during the hot deformation process. Figure 4 illustrates the grain orientations of AA7075 alloy under the influence of a strain rate of 0.01 s^−1^, at different temperatures of 350, 400, and 450 °C. As shown in Figure 4, compared with the grain morphology in the as-received samples, the increase in strain rate and deformation temperature will lead to different trends in grain morphology. It is noted that the impact of the deformation temperature on the microstructural evolution of the AA7075 alloy with the same strain rate of 0.01 s^−1^ is well described in Figure 4b–d. It can also be observed that as the temperature rises, the formation of new granules becomes more significant. The increasing number of recrystallized granules with increasing temperature indicates that DRX is more likely to occur at higher temperatures. The effect of the strain rate on the microstructural evolution of the AA7075 alloy at the same temperature value of 450 °C is shown in Figure 4d–f. It is fascinating to note that there are considerably more small grains at strain rates of 0.01 s^−1^ and 0.1 s^−1^ than there are at 1 s^−1^.

**Figure 5:**
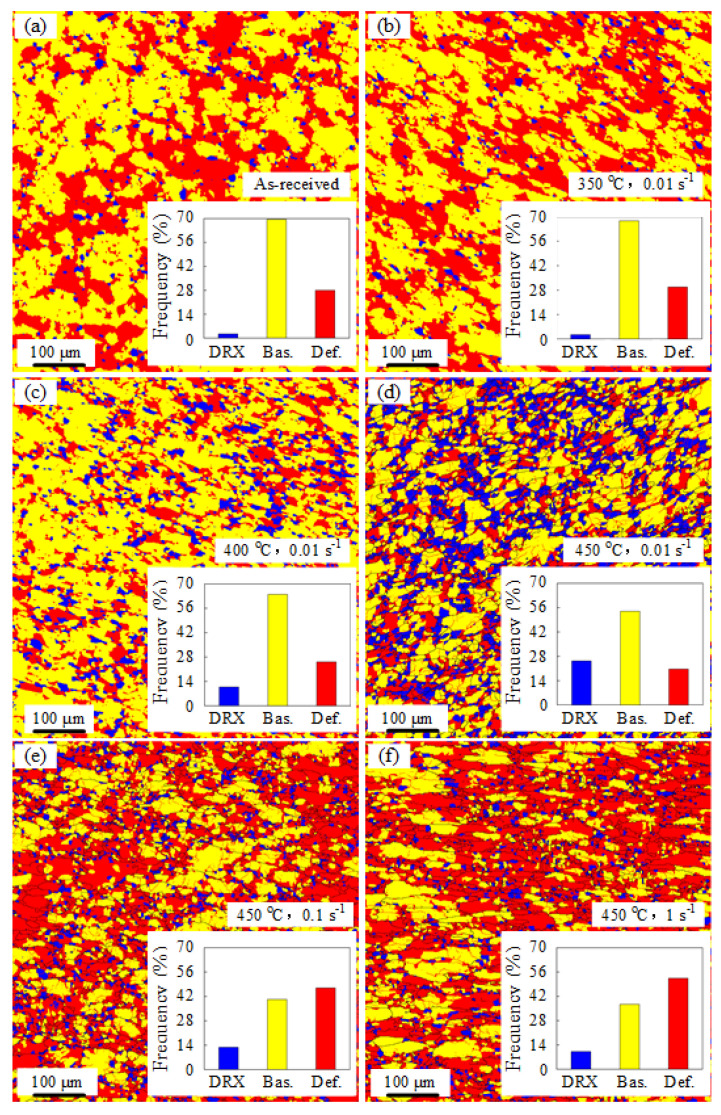
Grain orientation spread maps under different deformation conditions: (**a**) as-received; (**b**) 350 °C, 0.01 s^−1^; (**c**) 400 °C, 0.01 s^−1^; (**d**) 450 °C, 0.01 s^−1^; (**e**) 450 °C, 0.1 s^−1^, and (**f**) 450 °C, 1 s^−1^. Bas. and Def. in each figure represent the undeformed and deformed grains, respectively.

Figure 5 shows the individual grain orientation spread (GOS) corresponding to the inverse pole figure (IPF) shown in Figure 4. In particular, it can be observed that blue grains with misorientations between 0° and 2° represent DRX grains. In addition, the yellow grains with misorientations between 2° and 5° express deformed grains, while the red ones with misorientations between 5° and 35° denote severely deformed grains, respectively [4]. Then, the volume fraction of the recrystallized grains under various deformation conditions could be extracted, as presented in Table 2. In Figure 5a, a very small proportion of DRX grains is found in the as-received AA7075 alloy, which was mainly caused by the heat treatment of the original sheet. In Figure 5b, the volume fraction of DRX grains is 0.04 at 350 °C and 0.01 s^−1^, which indicates that there is almost no recrystallization although all these grains have undergone severe plastic deformation. With deformation temperature from 400 to 450 °C at the same strain rate of 0.01 s^−1^, the proportion of the recrystallized grains increased from 0.108 to 0.254, as illustrated in Figure 5c,d. Particularly, the volume fraction of DRX reached the peak value of 0.254 at 450 °C, indicating that the degree of DRX was comparatively lower than that of the deformed grains during the hot-stretching process. This could be attributed to the fact that the temperature rise was favorable for the DRX grain formation.

As can be ascertained from Figure 5d,e, at a strain rate of 0.1 s^−1^, the volume fraction of recrystallized grains decreased compared with that at a strain rate of 0.01 s^−1^. However, few DRX grains existed and severely deformed grains accounted for the vast majority of the deformed region since the strain rate increased to 1 s^−1^, as depicted in Figure 5f. Consequently, a lower strain rate during deformation led to the formation of a higher number of DRX grains provided the temperature was kept constant.

**Figure 6:**
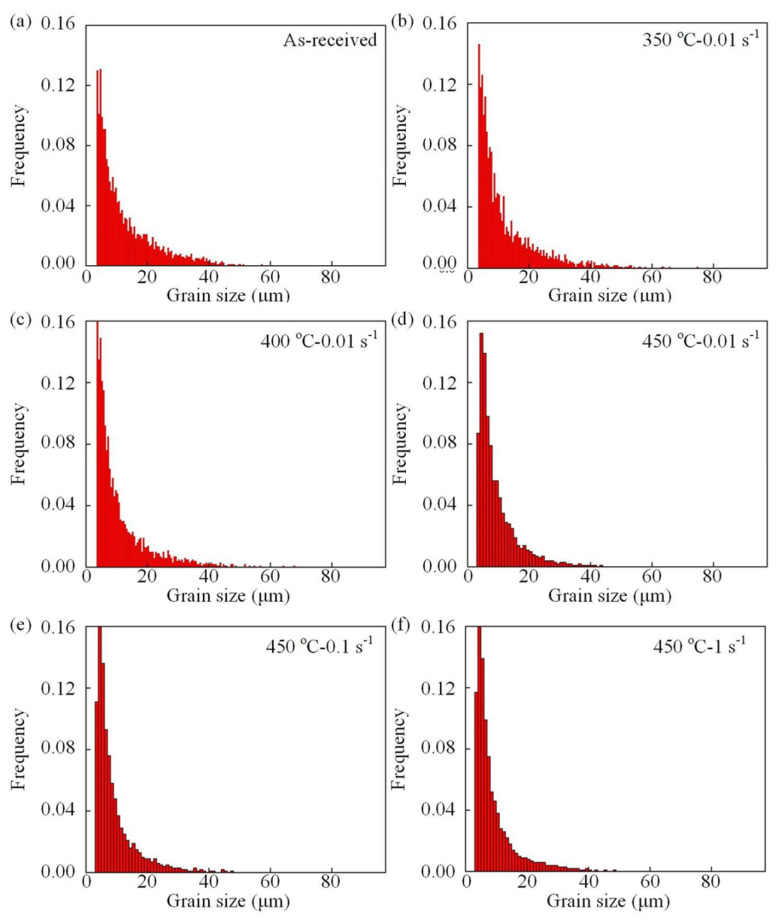
Comparison of grain size under different hot deformation conditions: (**a**) as-received; (**b**) 350 °C, 0.01 s^−1^; (**c**) 400 °C, 0.01 s^−1^; (**d**) 450 °C, 0.01 s^−1^; (**e**) 450 °C, 0.1 s^−1^, and (**f**) 450 °C, 1 s^−1^.

To further clarify the grain size evolution of the AA7075 alloy caused by DRX and the severe hot plastic deformation, the statistical frequency of the grain size under various hot deformation conditions is expressed in Figure 6. Meanwhile, extract the data in Figure 6 as shown in Table 3. The extracted outcomes show that the typical grain size exhibited a declining tendency as the deformation temperature was raised. This is because as the deformation temperature increased, the degree of recrystallization of the grains gradually increased, and the original grains at the grain boundaries disappeared, forming new and smaller grains. Moreover, when the deformation temperature was fixed at 450 °C, the average grain size experienced a marginal rise from 9.7 to 10.2 μm upon the strain rate increasing from 0.01 to 1 s^−1^. This was because the subgrains did not have sufficient time to form high-angle grain boundaries at a 1 s^−1^ due to the relatively short deformation period. Since DRX did not fully occur, thermal effects and DRX had negligible impact on the grain size.

### 3.2. Dislocation Evolution and Its Interaction with Precipitates

**Figure 7:**
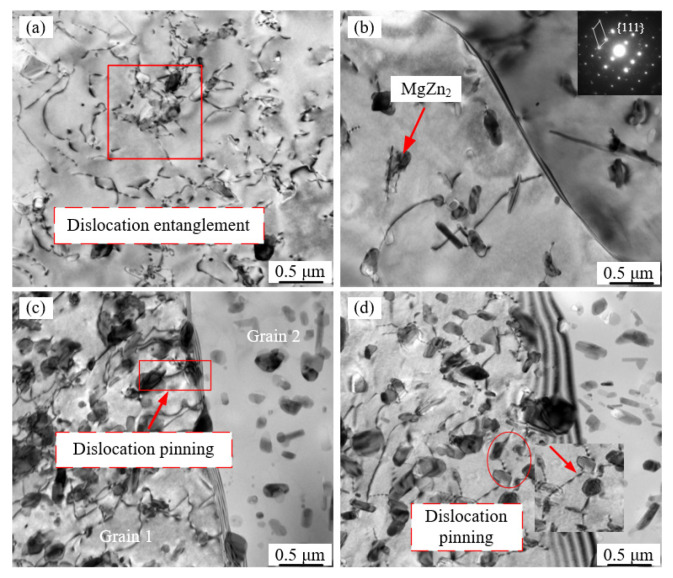
TEM characteristics of the AA7075 alloy at distinct conditions: (**a**) 350 °C, holding for 10 min; (**b**) 350 °C, 0.01 s^−1^; (**c**) 400 °C, holding for 10 min; and (**d**) 400 °C, 0.01 s^−1^.

During the holding and stretching processes at different temperature values, the precipitates were generated from the supersaturated solid solution (the so-called precipitate generation) as illustrated in Figure 1b. Meanwhile, a large number of dislocations associated with the evolution of the precipitates occurred due to severe plastic deformation. The impact of dislocations on the evolution of precipitates was revealed in depth using TEM analysis to characterize large numbers of intra- and intergranular dislocations that are associated with dynamic precipitates under different temperature values of 350 °C and 400 °C at 0.01 s^−1^, as displayed in Figure 7. Dislocation entanglement was observed in Figure 7a. At the same time, it can be seen that the elliptical precipitated phase is entangled with intra- and intergranular dislocations. It was found that the intermetallic compounds of AA7075 alloy precipitated by supersaturated solid solution mainly include η (MgZn_2_), T (Al_2_Mg_3_Zn_3_), and S (Al_2_CuMg) phases [32,33,34]. In this study, it should be noted that the elliptical phases were calibrated and determined as the η (MgZn_2_) phase, as shown in Figure 7b. In particular, there was a strong interaction between the precipitates and dislocations including the classic dislocation cutting mechanisms. In Figure 7c,d, both intergranular and intragranular dislocations were found to be pinned by smaller precipitates [20].

**Figure 8:**
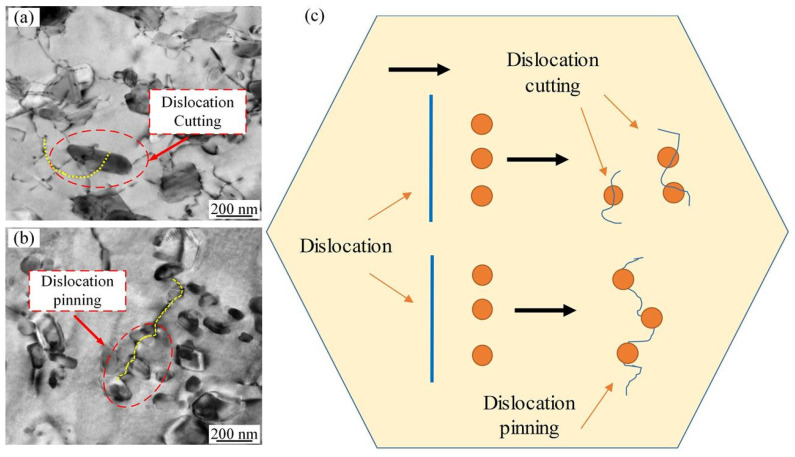
Interplays between dislocations and precipitates: (**a**) dislocation cutting; (**b**) dislocation pinning; (**c**) schematic diagram.

The remarkable interplays between dislocations and precipitates denote the existence of dislocations cutting and pinning with precipitates according to the TEM observation. Figure 8 depicts a schematic diagram of the above-mentioned mechanisms. It is noteworthy that the precipitates cut by dislocations lead to the formation of smaller precipitates. In addition, these precipitates promote further dislocation pinning to a certain extent.

### 3.3. Evolution of Microvoid Damage Induced by Precipitates

**Figure 9:**
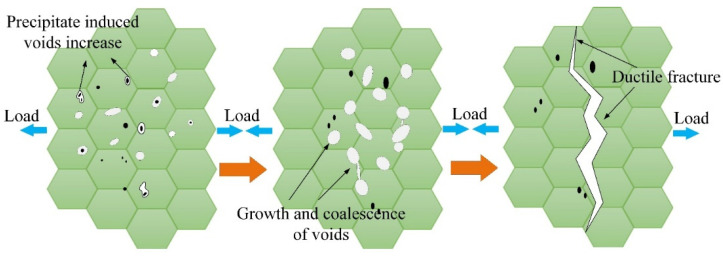
Schematic illustration of damage evolution during the application of uniaxial hot stretching. The formation of precipitation-induced voids, growth and coalescence, and ductile fracture are mainly included.

Therefore, these smaller precipitates hinder the movement of dislocations as well. Similarly, these tiny precipitates were crucial for the formation, growth, and coalescence of microvoids. Particularly, during the process of hot plastic deformation, the refined and hard precipitated phases were easily regarded as sites for void nucleation, which promoted the formation of microvoids, as has been confirmed by Simsek et al. [35]. Then, the nucleation and growth of microvoids subjected to external loads accelerated the generation and deterioration of microcracks, as can be observed in Figure 9.

**Figure 10:**
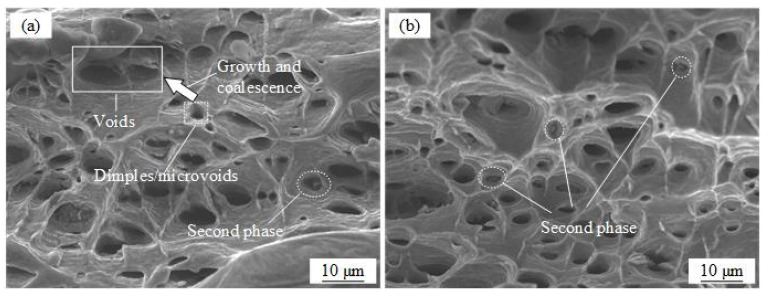
Fracture characterization at various strain rates and temperature values: (**a**) 350 °C and 0.1 s^−1^; (**b**) 350 °C and 1 s^−1^.

In order to analyze how the damage evolved of AA7075 alloy during hot plastic deformation, SEM micrographs characterizing the damage evolution of tensile specimens at 0.1 and 1 s^−1^ are shown in Figure 10. By increasing the strain rate, small and deep dimples were observed at 350 °C, which indicated ductile fracture. As can be observed from Figure 10a, a large number of microvoids accumulated to form voids at a strain rate of 0.1 s^−1^, which explained the formation of macroscopic cracks leading to ductile fracture. At the temperature value of 350 °C, the number of dimples increased rapidly with the strain rate increasing from 0.01 to 1 s^−1^. In addition, the second phase between or inside dimples was regarded as the nucleation sites for microvoids, as shown in Figure 10b. As a result, the microvoid-based damage evolution caused a decline in ductility [36,37].

## 4. Multiscale Modelling of Viscoplastic Behavior

In this section, an improved multiscale viscoplastic constitutive model was developed. This model emphasized the impact of precipitates and dislocations on the evolution of microvoid-based damage.

### 4.1. Damage Modelling Considering Precipitate Evolution

The irregularly shaped precipitates in the AA7075 alloy can promote ductile fracture resulting from internal defects [38]. Since damage evolution is a continuous process, it is important to note that microvoid nucleation plays a crucial role in initiating the successive growth and coalescence stages. Furthermore, the result of plastic strain rate and grain size on the microvoid nucleation rate is dominant. Therefore, the nucleated rate of microvoids ${\dot{D}}_{N_{r}}$ is given as follows [39]:

(1) $${\dot{D}}_{N_{r}}=\eta_{2}{\bar{d}}^{-\gamma_{5}}exp\left(\eta_{3}\varepsilon_{p}\right){\dot{\varepsilon }}_{p}^{d_{2}}$$

where $\eta_{2}$, $\eta_{3}$, and $\gamma_{5}$ are temperature-dependent parameters, $\bar{d}$ denotes the normalized grain size,$\;\varepsilon_{p}$ represents the plastic strain generated by the material during thermoplastic deformation, $d_{2}$ refers to a material constant, and ${\dot{\varepsilon }}_{p}$ illustrates the plastic strain rate.

However, the growth and further coalescence of microvoids may cause the gradual deterioration. The plastic strain rate, which varies with temperature, has a significant influence on damage evolution [5], and the growth rate of microvoids ${\dot{D}}_{G_{r}}$ can be described as follows:

(2) $${\dot{D}}_{G_{r}}=\eta_{1}\left(1-D\right){\dot{\varepsilon }}_{p}^{d_{1}}$$

where $\eta_{1}$ and $d_{1}$ are temperature-dependent constants, and D illustrates the overall damage evolution.

During hot plastic deformation, damage accumulation can result in macroscopic fracture closure due to grain rotation and accompanying grain boundary slip [40]. Thus, to precisely model damage evolution, the damage recovery rate ${\dot{D}}_{R_{r}}$ was proposed [38], which can be expressed as follows:

(3) $${\dot{D}}_{R_{r}}=-\eta_{4}D$$

where $\eta_{4}$ is a material parameter that varies with temperature and represents the combined effects of grain boundary slip and grain rotation. By taking into account the combined influence of ${\dot{D}}_{N_{r}}$, $\;{\dot{D}}_{G_{r}}$,and ${\dot{D}}_{R_{r}}$, the overall damage evolution rate can be mathematically represented as:

(4) $$\dot{D_{r}}=\eta_{1}\left(1-D\right){\dot{\varepsilon }}_{p}^{d_{1}}+\eta_{2}{\bar{d}}^{-\gamma_{5}}exp\left(\eta_{3}\varepsilon_{p}\right){\dot{\varepsilon }}_{p}^{d_{2}}-\eta_{4}D$$

By considering the microvoid-based damage, the flow stress $\sigma_{R}$ is adjusted to the effective stress σ/(1−D). Therefore, the plastic strain rate ${\dot{\varepsilon }}_{p_{r}}$ can be expressed as follows:

(5) $${\dot{\varepsilon }}_{p_{r}}={\left(\frac{\frac{\sigma }{\left(1-D\right)}-H-k}{K}\right)}^{n_{1}}{\left(\bar{d}\right)}^{-u}$$

The flow stress $\sigma_{R}$ is shown in Equation (6):

(6) $$\sigma_{R}=E\left(\varepsilon_{T}-\varepsilon_{p}\right)\left(1-D\right)$$

Kowalewski et al. [41] proposed a model to introduce the strengthening mechanism of the multi-precipitate phase at elevated temperature values. In the CDM framework, key strengthening and degradation of precipitates related to their volume fraction were revealed. On this basis, Murchú et al. [42] modified and proposed a plastic flow law in the hyperbolic sinusoidal form:

(7) $${\dot{\varepsilon }}_{cr}={\dot{\varepsilon }}_{0}exp\left(\frac{-\Delta F}{k_{B}T}\right)\sin h\left(\frac{\sigma \left(1-H\right)}{\sigma_{0}\left(1-D_{P}\right)\left(1-D_{cr}\right)}\right)\;$$

where ${\dot{\varepsilon }}_{0}$ is a constant denoting the pre-exponential creep, ΔF stands for the Helmholtz free energy, T is the absolute temperature, $k_{B}$ represents the Boltzmann constant, and σ signifies the applied stress. The state variables H, $D_{P}$, and $D_{cr}$ are associated with primary hardening, damage caused by precipitate coarsening, and intergranular cavitation, respectively.

Consequently, it should be noted that precipitate-induced damage also plays a comprehensive role. Simsek et al. [35] believed that the fine and hard precipitated phases are easily used as the location of void nucleation, thus promoting the formation of microvoids. From the fracture morphology in Figure 10a,b, there are precipitated phases with inclusions in the dimples [43,44]. Therefore, the damage caused by precipitate evolution $D_{P}$ is introduced by plastic strain, and Equation (5) can be modified as shown:

(8) $${\dot{\varepsilon }}_{p_{r}}={\left(\frac{\frac{\sigma }{\left(1-D\right)\left(1-D_{P}\right)}-H-k}{K}\right)}^{n_{1}}{\left(\bar{d}\right)}^{-u}$$

Accordingly, the rheological stress should be transformed into the following form:

(9) $$\sigma_{R}=\left(1-D\right)\left(1-D_{P}\right)E\left(\varepsilon_{T}-\varepsilon_{p}\right)$$

To further illustrate the impact of sediment evolution on damage, the damage $D_{P}$ caused by sediment coarsening is described as a function of the ratio of the initial average precipitates spacing $\lambda_{0}$ to the actual average precipitates spacing $\lambda_{\Omega }$:

(10) $$D_{P}=1-\frac{\lambda_{0}}{\lambda_{\Omega }}$$

where Ω represents the precipitated phase (MgZn_2_) in the Al-Zn-Mg alloy. The average spacing of precipitates can be expressed as a function of the diameter and volume fraction of precipitates, as follows:

(11) $$\lambda_{\Omega }=P_{\Omega }\left[{\left(\frac{\pi }{6f_{\Omega }}\right)}^{1/3}-1\right]$$

where $P_{\Omega }$ and $f_{\Omega }$ denote the size and volume fraction of the precipitates, respectively.

To account for strain-induced precipitate spacing, the evolution of the mean precipitate size is given as follows [45]:

(12) $$P_{\Omega }^{3}-P_{\Omega ,0}^{3}=K_{\Omega }t+\phi_{\Omega }\varepsilon_{cr}$$

where $P_{\Omega ,0}$ is the initial size of precipitate, $K_{\Omega }$ stands for the coarsening rate at elevated temperature, and $\emptyset_{k}$ is a constant representing the precipitate coarsening. It was assumed that the volume fraction of precipitates remains invariable in this work. Therefore, using Equation (10), the precipitation damage rate is given as follows [46]:

(13) $$\dot{D_{P}}=\frac{1}{3}\frac{P_{\Omega ,0}^{2}}{P_{\Omega }^{5}}\left(K_{\Omega ,v}+\emptyset_{\Omega }{\dot{\varepsilon }}_{cr}\right)\frac{1}{1-D_{P}}$$

where $\dot{D_{P}}$ is the precipitate damage rate, $K_{\Omega ,v}$ is the coarsening rate at elevated temperature, $\emptyset_{\Omega }$ represents the precipitate coarsening constant. From Figure 7b, it can be seen that the precipitates in hot-deformed AA7075 alloy mainly consists of MgZn_2_. Therefore, Equation (13) can be simplified as follows [42]:

(14) $$\dot{D_{P}}=\left(\frac{K_{v}+\emptyset {\dot{\varepsilon }}_{cr}}{3P_{0}^{3}}\right){\left(1-D_{P}\right)}^{3/2}$$

where $K_{v}$ is the thermal coarsening rate, ∅ denotes the coarsening state variables of the precipitates, and $P_{0}$ refers to the initial diameter of the precipitates.

The above-mentioned Equations (12)–(14) were established based on the kinetics of precipitate evolution in the aging stage [42]. In particular, there is no aging in the hot deformation process of AA7075 alloy. Consequently, the precipitates’ coarsening cannot be considered. In this study, it was assumed that coarsening state variables of the precipitates ∅=0. Equation (15) can be simplified to a more intuitive form [39,47]:

(15) $$\dot{D_{P}}=\left(\frac{K_{v}}{3P_{0}^{3}}\right){\left(1-D_{P}\right)}^{3/2}$$

where $K_{v}$ is the thermal coarsening rate.

### 4.2. Mesoscale Grain Size Evolution

The evolution rate of grain size can be described with utilized normalized grain size $\bar{d}=d/d_{0}$ [48,49]:

(16) $$\dot{\bar{d_{r}}}=w_{1}{\left(\bar{d}\right)}^{-\gamma_{1}}+w_{2}{\dot{\varepsilon }}_{p}{\left(\bar{d}\right)}^{-\gamma_{2}}-w_{3}{\dot{S}}^{\gamma_{3}}{\left(\bar{d}\right)}^{\gamma_{4}}$$

where $w_{1}$, $w_{2}$, $w_{3}$, $\gamma_{1}$, $\gamma_{2}$, $\gamma_{3}$, and $\gamma_{4}$ indicate temperature-dependent parameters. The equation comprises three terms. The first term represents the impact of thermal effects on grain growth, the next term describes the grain boundary sliding on grain growth, and the third term describes the decrease in grain size following DRX [15].

Taking the dislocation density and plastic strain rate into account, the evolution of the volume fraction of DRX can be given as follows [50]:

(17) $$\dot{S_{r}}=q_{1}\left[x\bar{\rho }-{\bar{\rho }}_{c}\left(1-S\right)\right]{\left(1-S\right)}^{q_{2}}{\dot{\varepsilon }}_{p}^{q_{6}}$$

where S is the DRX volume fraction, $q_{1}$,$\;q_{2}$, and $q_{6}$ are defined as parameters related to temperature. Additionally, $\bar{\rho }$ denotes the dislocation density normalized by its initial value, while ${\bar{\rho }}_{c}$ signifies the threshold dislocation density required for DRX to occur.

### 4.3. Microscale Dislocation Density Evolution

Apart from the mesoscale grain size and damage evolution, the microscale evolution of dislocation density is crucial in the strain hardening and dynamic softening during hot plastic deformation. The evolution rate of dislocation density was expressed using normalized dislocation density $\bar{\rho }$ is given [51,52]:

(18) $$\dot{\bar{\rho_{r}}}=A\left(1-\bar{\rho }\right)\left|{\dot{\varepsilon }}_{p}\right|-K_{1}{\bar{\rho }}^{\delta_{1}}$$

where A and $K_{1}$ represent parameters related to the temperature, and $\delta_{1}$ denotes the material constant. In Equation (18), the first term refers to the dislocation accumulation during the plastic deformation procedure, which represents the strain hardening effect, while the second term refers to the reduction of dislocations induced by dynamic recovery.

### 4.4. Parameter Calibration

To summarize, a set of viscoplastic models were proposed emphasizing the combined impact of dislocations and precipitates for AA7075 alloy, as shown in Equation (19). To calibrate the material parameters based on the stress–strain curves derived by uniaxial hot stretching of the AA7075 alloy, they were classified into macroscopic state variables, such as strain rate and microscopic damage, grain size, dislocation density, and precipitate-induced damage [53,54]. The detailed calibration and optimization methods based on the genetic algorithm were described in our previous work [31]. Table 4 presents the calibrated values of the material parameters. We presented the temperature-dependent parameters in our previous work, where T represented the absolute temperature. The activation energy for the corresponding material constants is denoted by the symbol *Q* with a subscript. Combining TEM observation and Image Pro Plus 6.0 analysis software, the average diameter of precipitate $P_{0}$ was obtained based on the size of the precipitate at three different temperatures of 350, 400, and 450 °C.

(19) $$\left\{\begin{array}{l} {\dot{\varepsilon }}_{p_{r}}={\left(\frac{\frac{\sigma }{\left(1-D\right)\left(1-D_{P}\right)}-H-k}{K}\right)}^{n}{\left(\overset{-}{d}\right)}^{-u}\\ \dot{\overset{-}{\rho_{r}}}=A{\overset{-}{d}}^{n_{1}}\left(1-\overset{-}{\rho }\right)\left|{\dot{\varepsilon }}_{p}\right|-K_{1}{\overset{-}{\rho }}^{\delta_{1}}-\frac{K_{2}\overset{-}{\rho }}{1-S}\dot{S}\\ \dot{S_{r}}=q_{1}\left[x\overset{-}{\rho }-{\overset{-}{\rho }}_{c}\left(1-S\right)\right]\times \left[{\left(1-S\right)}^{q_{2}}\right]{\dot{\varepsilon }}_{p}^{q_{6}}\\ \dot{x}=q_{5}\left(1-x\right)\overset{-}{\rho }\\ {\overset{-}{\rho }}_{c}=q_{3}{\dot{\varepsilon }}_{p}^{q_{4}}\\ H=B{\overset{-}{\rho }}^{0.5}\\ \sigma_{f}=\left(1-D\right)(1-D_{P})E\left(\varepsilon_{T}{-\varepsilon }_{p}\right)\\ \dot{\overset{-}{d_{r}}}=w_{1}{\left(\overset{-}{d}\right)}^{{-\gamma }_{1}}+w_{2}{\dot{\varepsilon }}_{p}{\left(\overset{-}{d}\right)}^{{-\gamma }_{2}}-w_{3}{\dot{S}}^{\gamma_{3}}{\left(\overset{-}{d}\right)}^{\gamma_{4}}\\ \dot{D_{r}}=\eta_{1}\left(1-D\right){\dot{\varepsilon }}_{p}^{d_{1}}+\eta_{2}{\overset{-}{d}}^{{-\gamma }_{5}}exp\left(\eta_{3}\varepsilon_{p}\right){\dot{\varepsilon }}_{p}^{d_{2}}-\eta_{4}D\\ \dot{D_{P}}=\left(\frac{K_{v}}{3P_{0}^{3}}\right){\left(1-D_{P}\right)}^{3/2} \end{array}\right.$$

## 5. Verification

**Figure 11:**
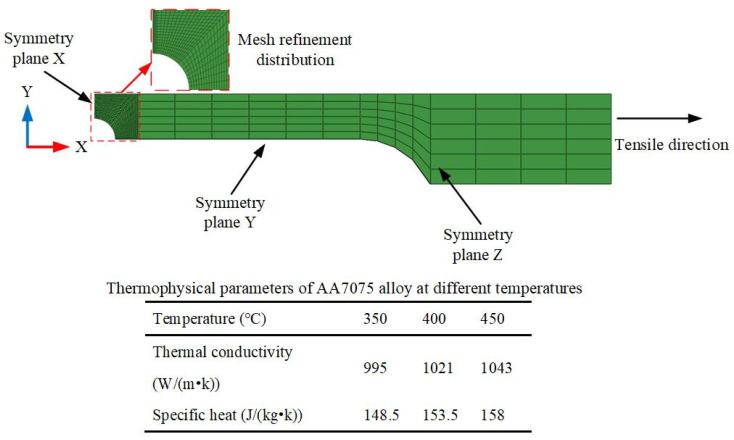
FE model of the notched specimens.

According to the symmetrical geometric characteristics of the specimens, the finite element model of one-eighth notched specimens was established as shown in Figure 11. The finite element model meshed with a total number of 6640 C3D8T elements, and the thickness direction meshed into 5 layers. To obtain accurate simulated results, the mesh of the central notched part was refined.

The proposed viscoplastic constitutive model coupled with damage evolution was implemented in Abaqus/Explicit through an in-house VUMAT subroutine. According to the temperature distribution during the hot tensile tests, a stable temperature field ranging from 350 to 450 °C was applied to the entire FE model.

**Figure 12:**
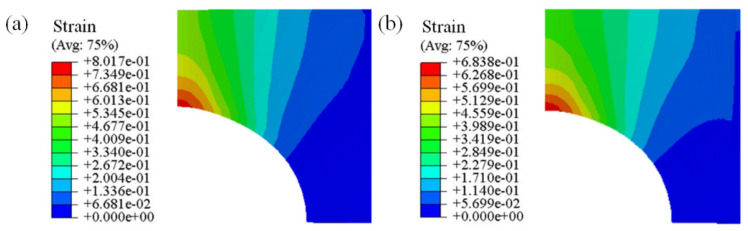
Fracture strain of notched specimens at different temperature values and strain rates: (**a**) 350 °C, 0.01 s^−1^; (**b**) 400 °C, 0.01 s^−1^.

Figure 12 shows the simulated fracture strain of the notched specimen at 350 and 400 °C with a strain rate of 0.01 s^−1^. In particular, the fracture strain was calculated according to $\varepsilon_{f}=\ln\left(\frac{A_{0}}{A_{f}}\right)$ [55], where $A_{0}$ refers to the initial region of the cross section, $A_{f}$ denotes the fractured cross-sectional area, and $\varepsilon_{f}$ is the fracture strain.

The comparison between the experimental results and simulated fracture strain is presented in Table 5. The results indicate that the simulated values accurately represent the ductile fracture behavior of the AA7075 alloy.

**Figure 13:**
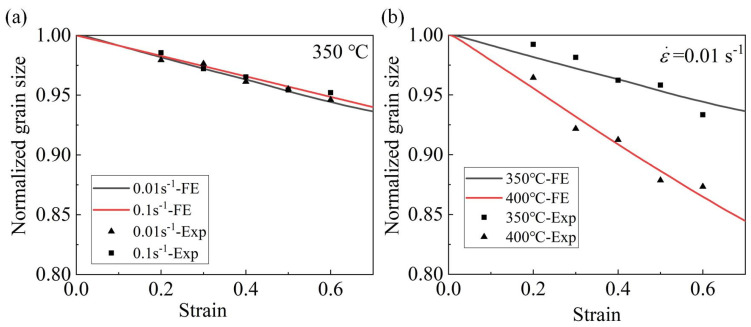
Comparison of grain size evolution of the AA7075 alloy at (**a**) 350 °C and (**b**) strain rate of 0.01 s^−1^.

A comparison between the experimental and simulated results of the normalized grain size of the AA7075 alloy is shown in Figure 13. The experimental normalized grain size was found to be in good agreement with the line predicted by the simulations, indicating the accuracy of the FE model in predicting the grain size evolution of the AA7075 alloy. As can be seen in Figure 13a, the normalized grain size decreased with the increasing deformation temperature at a strain rate of 0.01 s^−1^ because DRX occurred at higher temperature values in the AA7075 alloy. Additionally, taking into account the data depicted in Figure 13b, it can be inferred that the normalized grain size was reduced by decreasing the strain rate at a deformation temperature of 350 °C.

**Figure 14:**
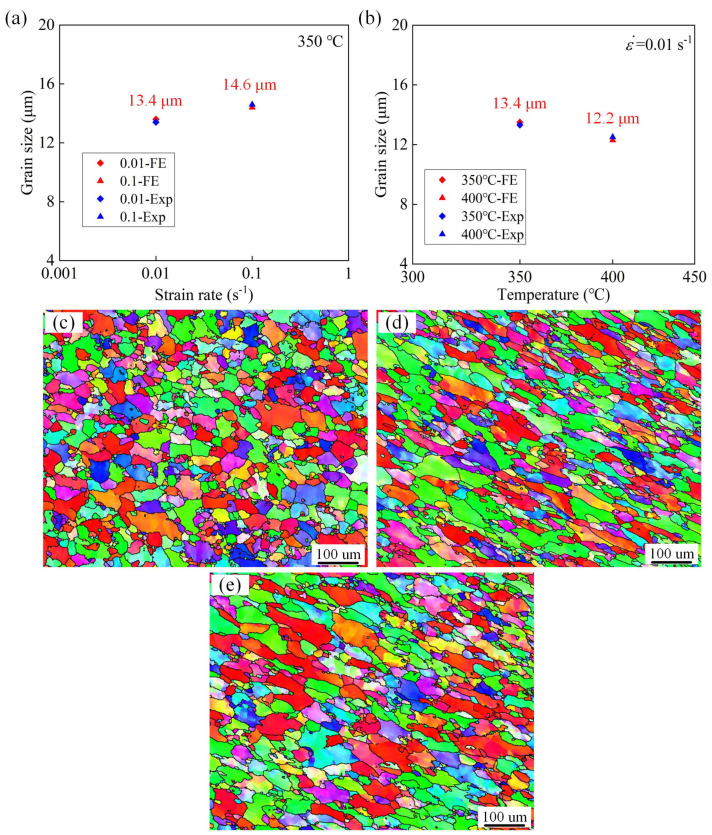
Evolution of the grain size at various temperatures and strain rates: (**a**) 0.01 s^−1^ and 0.1 s^−1^ with a temperature of 350 °C; (**b**) 350 °C and 400 °C with a strain rate of 0.01 s^−1^. Morphology of grain size at the scenarios of (**c**) 350 °C-0.01 s^−1^, (**d**) 350 °C-0.1 s^−1^, and (**e**) 400 °C-0.01 s^−1^.

To further verify the reliability of evolution of grain size, the average grain size of the notched samples deformed under various scenarios was calculated in Figure 14. The experimental values of the average grain size are relatively close to the simulated values, which further proves the accuracy in predicting the grain size evolution. More specifically, as can be ascertained from Figure 14a, when the deformation temperature is 350 °C, the measured average grain size is 13.4 μm (shown in Figure 14c) at a strain rate of 0.01 s^−1^. In addition, a similar grain size of 14.6 μm (shown in Figure 14d) is obtained at a strain rate of 0.1 s^−1^. The grain size increases slightly, but not significantly, with the increasing strain rate. This is mainly because the thermal effect is less influential than that of DRX. Figure 14b illustrates that at the strain rate is 0.01 s^−1^, the average grain size in the experiment is 13.4 μm at 350 °C and 12.2 μm (shown in Figure 14e) at 400 °C, suggesting that increasing the temperature leads to a gradual decrease in the average grain size. This reduction is attributed to the occurrence of DRX during the thermal deformation process of the AA7075 alloy, which occurs more frequently at higher temperatures.

## 6. Multiscale Investigation of Defect Formation in Hot-Formed U-Parts

Actually, the damage evolution and heterogeneous thickness distribution are crucial issues restricting formability. In this section, a multiscale investigation was conducted including precipitate-induced damage, and the macroscopic thickness distribution of hot-formed U-parts was predicted.

### 6.1. FE Setup of U-Shaped Parts in the Hot-Forming Process

**Figure 15:**
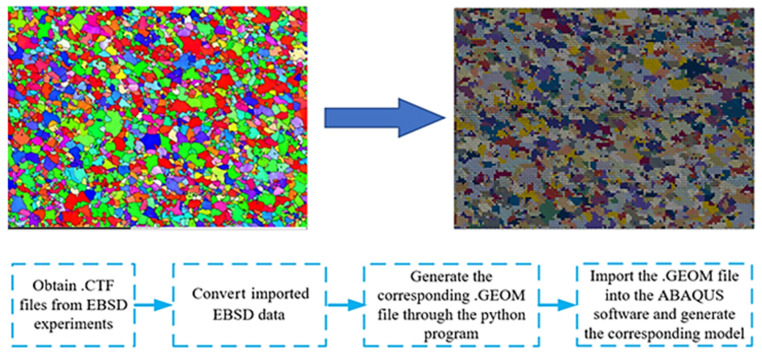
Reconstruction of the virtual representative volume element (RVE) model of the AA7075 alloy sheet.

**Figure 16:**
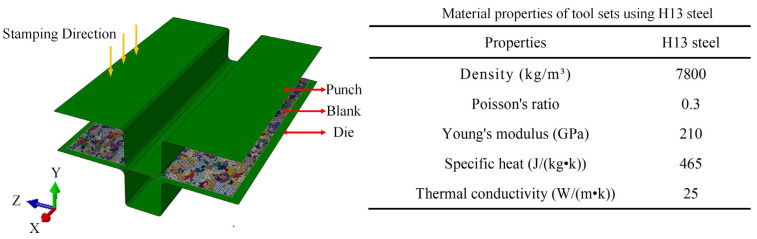
FE model of the hot U-stamping process.

To input the initial grain size before hot plastic deformation into the FE model, the actual grain morphology from EBSD observation was reconstructed based on in-house Python codes, as displayed in Figure 15. The FE model of the U-forming process consists of the punch, die, and blank, as shown in Figure 16. To improve the calculation efficiency, shell elements were also employed in the blank. The punch and die were defined as rigid bodies, and the initial temperature was set to 25 °C. In addition, the blanks were given deformation temperature values of 350, 400, or 450 °C during the hot-forming process. The stamping speed of the punch in the hot-forming process was set to 20 mm/s, and the friction coefficient between the blank and die was set to 0.1.

### 6.2. Damage Distribution

**Figure 17:**
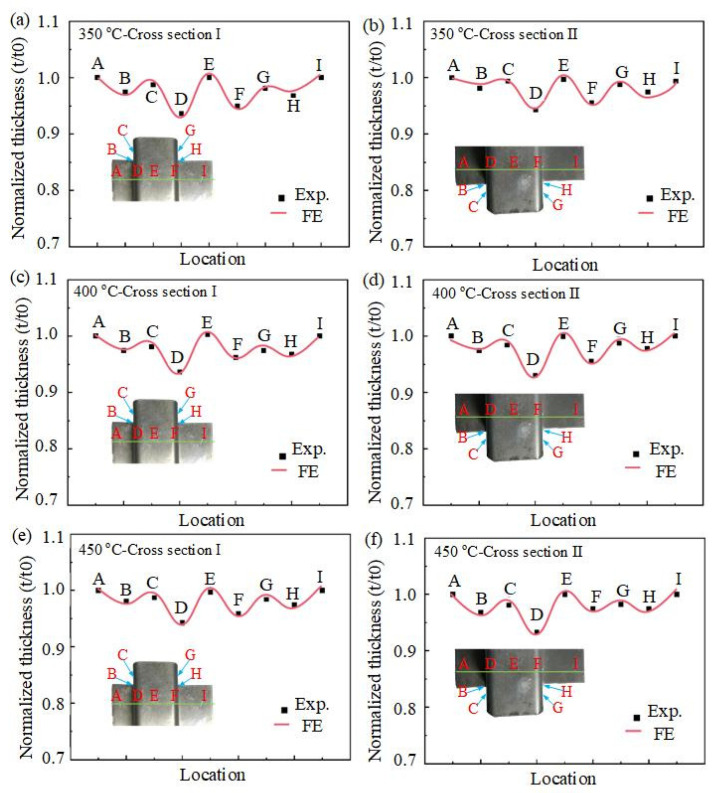
Thickness distributions of the U-shaped components from experiments (symbols) and simulations (curves).

To investigate the precision of the forming process, the normalized thickness distribution ($t/t_{0}$) of the U-shaped components, which contained angular features, was manually measured using Vernier calipers. Specifically, two cross-sectional positions were chosen from the hot-formed U-parts, and nine points were measured and extracted for each section to validate the simulation accuracy. Figure 17 shows the thickness distribution of the hot-formed U-shaped parts obtained through the FE model at different deformation temperatures.

The simulated and experimental results demonstrate that excellent agreement at the temperature values of 350, 400, and 450 °C was attained, with a maximum deviation in normalized thinning of less than 5%. The degree of local thinning in the corner region was similar for all temperature values. Compared with other positions, the thicknesses of the parts at marked points D and F were the thinnest, which occurred in both the front and back parts. This is because the thick-walled U-shaped parts were typical deep-drawing parts. During the stamping process, the sheet metal slid into the cavity of the concave mold against the fillet of the die, and the flow of the sheet material at the fillet of the die was blocked, resulting in tensile stress being generated on the sidewall. During the forming process, the cooling shrinkage effect caused the sheet material to generate thermal stress. This situation further aggravated the tendency to crack, resulting in the thinning of the material at the rounded corners of the die. Therefore, it can be judged that the heterogeneous plastic strain mainly occurred at the corner of the U-shaped parts.

**Figure 18:**
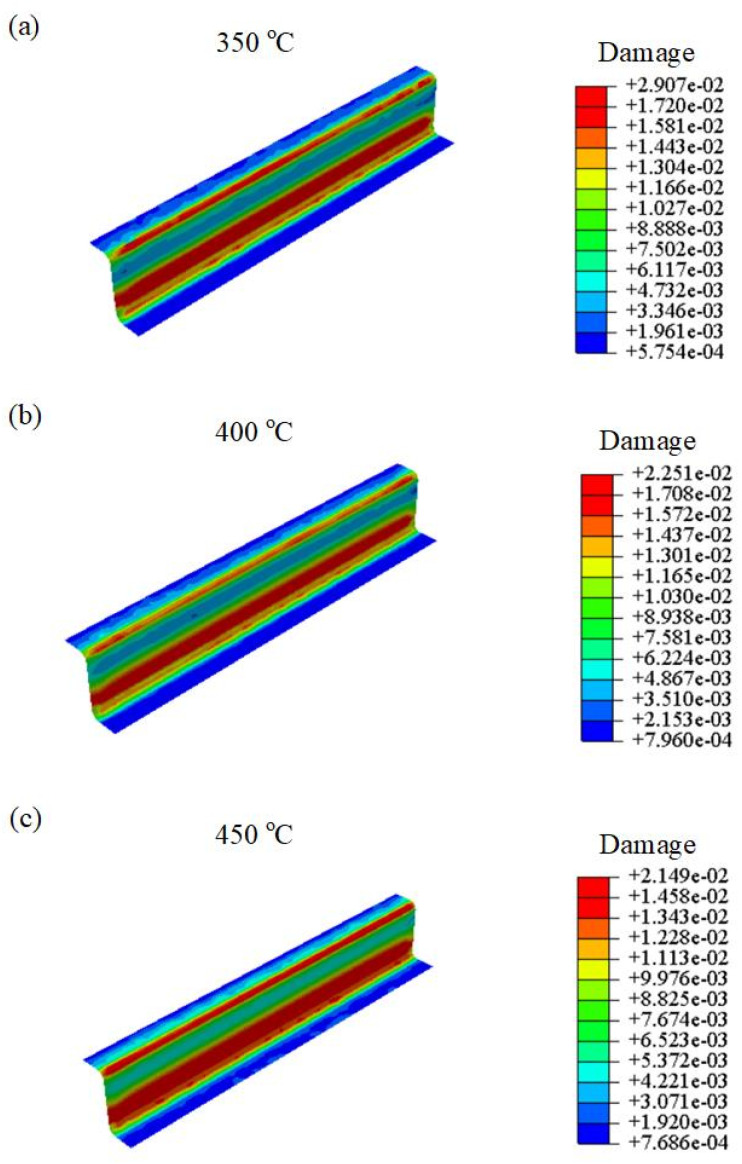
Damage distributions of the U-shaped components at various temperature values from (**a**) 350 °C, (**b**) 400 °C to (**c**) 450 °C.

Figure 18 shows the predicted damage distribution of U-shaped specimens at different stamping temperature values. It can be seen that the damage is mainly distributed in the bending part. By increasing the deformation, the plastic strain and damage within the bending positions increased gradually. Particularly, at the temperature value of 350 °C, the maximum damage of U-shaped parts was 0.029. As the temperature increased to 400 °C, the value of damage decreased to 0. 0225. Furthermore, at the temperature value of 450 °C, the value of the damage was reduced to 0.0214. Therefore, under the same deformation, the damage value of U-shaped parts decreased by increasing the temperature. It is worth mentioning that Figure 18 mainly predicts the concentrated damage areas of U-shaped parts during the hot-stamping process. In the future, SEM tests will be performed on the damaged areas of the samples to determine the existence of voids, thereby further verifying the accuracy of damage prediction.

**Figure 19:**
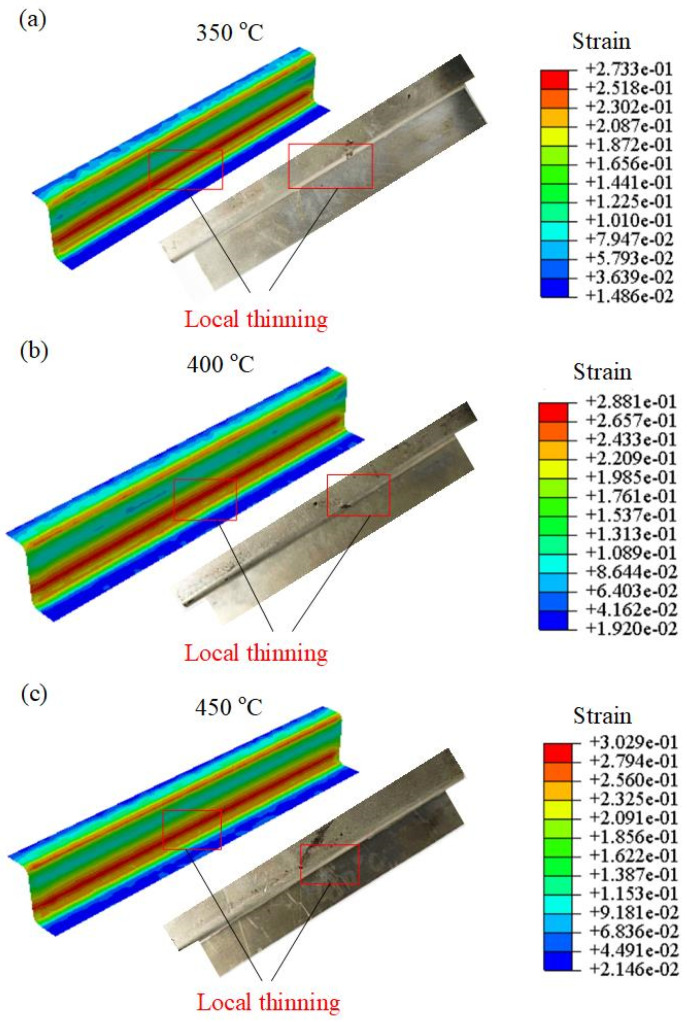
Plastic strain distributions of the U-shaped components at distinct temperatures from (**a**) 350 °C, (**b**) 400 °C to (**c**) 450 °C.

At a constant stamping speed of 20 mm/s, the evolution of the plastic strain of the U-shaped parts at deformation temperatures of 350 °C, 400 °C, and 450 °C is shown in Figure 19. The largest plastic strain occurred in the corner region, which is called local thinning, and was caused by the fact that the plastic flow of the center area is more uniform than that of the corner. Moreover, the damage evolution at 450 °C demonstrated consistency with that observed at 350 °C and 400 °C. Consequently, the proposed model exhibited significant advantages in predicting the damage evolution of hot-stamped parts.

## 7. Conclusions

In this study, the uniaxial tensile tests of AA7075 alloy at elevated temperatures have been conducted, and subsequently, the influence of the interaction between precipitates and dislocations on the damage evolution based on microvoids through TEM and SEM has been characterized. During thermoplastic deformation, the elliptical η (MgZn_2_) phase occupies the largest proportion and is entangled with intragranular and intergranular dislocations. Specifically, there is a strong interaction between the coarse precipitates and dislocations, including the cutting and bypassing mechanisms of dislocations. In addition, these fine precipitates induce the formation of microvoids, which then accelerate the generation of microcracks. Based on this mechanism, an improved multiscale viscoplastic model that emphasizes the effects of precipitates and dislocations on the evolution of damage based on microvoids has been established. Then, the established model has been validated by comparing the fracture strain and grain size of experimental and simulation results.

In addition, a multiscale study focusing on the plastic strain distribution and damage evolution of hot-formed U-shaped parts has been further analyzed. In the process of hot plastic deformation, the cumulative rate of damage gradually decreases with the increase in strain rate and increases with the increase in deformation temperature. In addition, the evolution of damage is closely related to the evolution of plastic strain, in which local thinning occurs due to the local plastic flow.

## Figures and Tables

**Table 1:** The composition of the as-received AA7075 alloy (w.t. %).

Composition	Zn	Cu	Mg	Fe	Cr	Mn	Ti	Si	Al
w.t./%	5.4	1.5	2.2	0.5	0.21	0.3	0.2	0.04	Bal.

**Table 2:** Volume fraction of the DRX grains under different temperatures and strain rates.

Temperature/°C	Strain Rate/s^−1^	Volume Fraction
350	0.01	0.04
400	0.01	0.108
450	0.01	0.254
450	0.1	0.123
450	1	0.096

**Table 3:** Average grain size at different temperatures and strain rates.

Temperature/°C	Strain Rate/s^−1^	Average Grain Size/μm
350	0.01	12.9
400	0.01	11.7
450	0.01	9.7
450	0.1	9.8
450	1	10.2

**Table 4:** Calibrated material parameters.

Material Parameters	Values	Material Parameters	Values
$k_{0}$ (MPa)	3.821 × 10^0^	$w_{30}$ (dimensionless)	2.075 × 10^0^
$Q_{k}$ (J/mol)	1.836 × 10^4^	$Q_{w3}$ (J/mol)	6.127 × 10^3^
$K_{0}$ (MPa)	2.512 × 10^0^	$\gamma_{10}$ (dimensionless)	2.607 × 10^0^
$Q_{K}$ (J/mol)	2.026 × 10^4^	$Q_{\gamma 1}$ (J/mol)	8.507 × 10^2^
$n_{10}$ (dimensionless)	8.524 × 10^−2^	$\gamma_{20}$ (dimensionless)	2.079 × 10^0^
$Q_{n1}$ (J/mol)	2.228 × 10^4^	$Q_{\gamma 2}$ (J/mol)	7.127 × 10^1^
$B_{0}$ (MPa)	4.853 × 10^0^	$\gamma_{30}$ (dimensionless)	9.209 × 10^−1^
$Q_{B}$ (J/mol)	1.379 × 10^3^	$Q_{\gamma 3}$ (J/mol)	9.497 × 10^2^
$A_{0}$ (dimensionless)	2.169 × 10^−3^	$\gamma_{40}$ (dimensionless)	1.355 × 10^−2^
$Q_{A}$ (J/mol)	2.774 × 10^4^	$Q_{\gamma 4}$ (J/mol)	1.187 × 10^4^
$K_{10}$ (dimensionless)	2.831 × 10^−2^	$\gamma_{50}$ (dimensionless)	2.769 × 10^−2^
$Q_{K1}$ (J/mol)	2.556 × 10^4^	$Q_{\gamma 5}$ (J/mol)	6.867 × 10^3^
$K_{20}$ (dimensionless)	1.638 × 10^−2^	$\eta_{10}$ (dimensionless)	8.487 × 10^−2^
$Q_{K2}$ (J/mol)	2.203 × 10^4^	$Q_{\eta 1}$ (J/mol)	9.438 × 10^0^
$q_{10}$ (dimensionless)	3.579 × 10^0^	$C_{\eta_{2}}$ (dimensionless)	1.812 × 10^−2^
$Q_{q1}$ (J/mol)	3.609 × 10^4^	$\eta_{20}$ (dimensionless)	8.548 × 10^10^
$q_{20}$ (dimensionless)	4.623 × 10^−1^	$Q_{\eta 2}$ (J/mol)	1.715 × 10^5^
$Q_{q2}$ (J/mol)	4.440 × 10^3^	$C_{\eta_{3}}$ (dimensionless)	6.320 × 10^−2^
$q_{30}$ (dimensionless)	6.026 × 10^−6^	$\eta_{30}$ (dimensionless)	7.217 × 10^−1^
$Q_{q3}$ (J/mol)	3.618 × 10^4^	$Q_{\eta 3}$ (J/mol)	1.237 × 10^4^
$q_{40}$ (dimensionless)	1.308 × 10^−1^	$\eta_{40}$ (dimensionless)	2.233 × 10^−4^
$Q_{q4}$ (J/mol)	1.335 × 10^3^	$Q_{\eta 4}$ (J/mol)	4.058 × 10^4^
$q_{50}$ (dimensionless)	2.108 × 10^1^	$E_{0}$ (MPa)	1.158 × 10^3^
$Q_{q5}$ (J/mol)	3.815 × 10^3^	$Q_{E}$ (J/mol)	7.460 × 10^3^
$q_{60}$ (dimensionless)	5.236 × 10^−2^	$d_{1}$ (dimensionless)	9.418 × 10^−1^
$Q_{q6}$ (J/mol)	5.445 × 10^3^	$d_{2}$ (dimensionless)	9.150 × 10^−1^
$w_{10}$ (dimensionless)	1.111 × 10^−7^	$n_{1}$ (dimensionless)	1.025 × 10^−1^
$Q_{w1}$ (J/mol)	1.875 × 10^3^	$b_{1}$ (dimensionless)	9.419 × 10^3^
$w_{20}$ (dimensionless)	2.180 × 10^−5^	u (dimensionless)	5.050 × 10^−1^
$Q_{w2}$ (J/mol)	2.528 × 10^2^	$P_{0}$ (μm)	0.179
$K_{v}$ (MPa^−3^h^−1^)	1.110 × 10^12^		

**Table 5:** Comparison of the fracture strains at the strain rate of 0.01 s^−1^.

Temperature/°C	Experiments	Simulations
350	0.804	0.807
400	0.681	0.683

## Data Availability

Not applicable.

## References

[1] Zhang W., Xu J. (2022). Advanced lightweight materials for Automobiles: A review. Mater. Des..

[2] Zhou Z.W., Gong H.Y., You J., Liu S., He J. (2021). Research on compression deformation behavior of aging AA6082 aluminum alloy based equation and PSO-BP network model. Mater. Today Commun..

[3] Hou R., Hu P., Liang Y., Hou W., Dai M. (2021). Modeling the anisotropic plasticity and damage of AA7075 alloy in hot forming. Int. J. Mech. Sci..

[4] Ying L., Gao T., Dai M., Hu P., Dai J. (2020). Towards joinability of thermal self-piercing riveting for AA7075-T6 aluminum alloy sheets under quasi-static loading conditions. Int. J. Mech. Sci..

[5] Dalai B., Moretti M.A., Åkerström P., Arvieu C., Jacquin D., Lindgren L.-E. (2021). Mechanical behavior and microstructure evolution during deformation of AA7075-T651. Mater. Sci. Eng. A.

[6] Li X., Xu M., Zhang Z. (2021). Hot damage evolution in a high strength aluminum alloy during hot forming: A study using the Gurson–Tvergaard–Needleman model. J. Mater. Res. Technol..

[7] Fincato R., Tsutsumi S. (2021). Coupled elasto-viscoplastic and damage model accounting for plastic anisotropy and damage evolution dependent on loading conditions. Comput. Methods Appl. Mech. Eng..

[8] Sérgio E.R., Antunes F.V., Neto D.M., Borges M.F. (2021). Study on the Influence of the Gurson–Tvergaard–Needleman Damage Model on the Fatigue Crack Growth Rate. Metals.

[9] Sunar T., Özyürek D. (2021). A Research on the Effect of Retrogression and Re-Aging Heat Treatment on Hot Tensile Properties of AA7075 Aluminum Alloys. J. Manuf. Sci. Eng..

[10] Scharifi E., Savaci U., Kavaklioglu Z., Weidig U., Turan S., Steinhoff K. (2021). Effect of thermo-mechanical processing on quench-induced precipitates morphology and mechanical properties in high strength AA7075 aluminum alloy. Mater. Charact..

[11] Liu F., Bai P.C., Hou X.H., Xing Y.M. (2016). Quantitative Measurement of Strain Field around η′ Phase in a 7000 Series Aluminum Alloy. Mater. Sci. Forum.

[12] Chen H., Wang Z., Xu X., Vitus T., Jiang Z., Mao Q., Wang H., Liu Q., Zhang X., Liu Y. (2019). Effect of solid solution heat treatment following age hardening on microstructure and mechanical properties of 7000 series power aluminum alloy. Mater. Res. Express.

[13] Zhang Z., Cui Y., Chen Q. (2022). Damage and failure characterization of 7075 aluminum alloy hot stamping. J. Mech. Sci. Technol..

[14] Rahmaan T., Noder J., Abedini A., Zhou P., Butcher C., Worswick M.J. (2019). Anisotropic plasticity characterization of 6000- and 7000-series aluminum sheet alloys at various strain rates. Int. J. Impact Eng..

[15] Peng G.S., Chen K.H., Chen S.Y., Fang H.C. (2015). Evolution of the second phase particles during the heating-up process of solution treatment of Al−Zn−Mg−Cu alloy. Mater. Sci. Eng. A.

[16] Zhou P., Song Y., Hua L., Lu J., Zhang J., Wang F. (2019). Mechanical behavior and deformation mechanism of 7075 aluminum alloy under solution induced dynamic strain aging. Mater. Sci. Eng. A.

[17] Murchú C., Leen S., O’donoghue P., Barrett R. (2018). A precipitate evolution-based continuum damage mechanics model of creep behaviour in welded 9Cr steel at high temperature. Proc. Inst. Mech. Eng. Part L J. Mater. Des. Appl..

[18] Ying L., Liu W.Q., Wang D.T., Hu P., Wang Q. (2016). Experimental and simulation of damage evolution behavior for 7075-T6 aluminum alloy in warm forming. Chin. J. Nonferrous Met..

[19] Han N.M., Zhang X.M., Liu S., He D., Zhang R. (2011). Effect of solution treatment on the strength and fracture toughness of aluminum alloy 7050. J. Alloys. Compd..

[20] Dixit M., Mishra R.S., Sankaran K.K. (2008). Structure–property correlations in Al 7050 and Al 7055 high-strength aluminum alloys. Mater. Sci. Eng. A.

[21] Saravanan L., Senthilvelan T. (2016). Constitutive equation and microstructure evaluation of an extruded aluminum alloy. J. Mater. Res. Technol..

[22] Lin Y., Chen X.-M., Liu G. (2010). A modified Johnson–Cook model for tensile behaviors of typical high-strength alloy steel. Mater. Sci. Eng. A.

[23] He A., Xie G.L., Zhang H.L., Wang X. (2013). A comparative study on Johnson–Cook, modified Johnson–Cook and Arrhenius-type constitutive models to predict the high temperature flow stress in 20CrMo alloy steel. Mater. Des..

[24] Rotpai U., Arlai T., Nusen S., Juijerm P. (2021). Novel flow stress prediction and work hardening behavior of aluminium alloy AA7075 at room and elevated temperatures. J. Alloys Compd..

[25] Wang Y., Liu K., Hu N., Wei Q., Qiao G., Yang C., Lang L. (2020). Viscoplastic constitutive equations for modeling fluid loading and damage evolution during warm medium forming. Eng. Fract. Mech..

[26] Lu J., Song Y., Hua L., Zheng K., Dai D. (2018). Thermal deformation behavior and processing maps of 7075 aluminum alloy sheet based on isothermal uniaxial tensile tests. J. Alloys Compd..

[27] Rong H., Hu P., Ying L., Hou W., Zhang J. (2019). Thermal forming limit diagram (TFLD) of AA7075 aluminum alloy based on a modified continuum damage model: Experimental and theoretical investigations. Int. J. Mech. Sci..

[28] Chen F., Qu H., Wu W., Zheng J.-H., Qu S., Zheng Y. (2021). A Physical-Based Plane Stress Constitutive Model for High Strength AA7075 under Hot Forming Conditions. Metals.

[29] Li Y., Yu L., Zheng J.-H., Guan B., Zheng K. (2021). A physical-based unified constitutive model of AA7075 for a novel hot forming condition with pre-cooling. J. Alloys Compd..

[30] Xiao W., Wang B., Wu Y., Yang X. (2018). Constitutive modeling of flow behavior and microstructure evolution of AA7075 in hot tensile deformation. Mater. Sci. Eng. A.

[31] Tang B., Li H., Guo N., Zhang H., Liu G., Li X., Zuo Y. (2021). Revealing ductile/quasi-cleavage fracture and DRX-affected grain size evolution of AA7075 alloy during hot stamping process. Int. J. Mech. Sci..

[32] Cong F.-G., Zhao G., Jiang F., Tian N., Li R.-F. (2015). Effect of homogenization treatment on microstructure and mechanical properties of DC cast 7X50 aluminum alloy. Trans. Nonferrous Met. Soc. China.

[33] Zou X.-L., Yan H., Chen X.-H. (2017). Evolution of second phases and mechanical properties of 7075 Al alloy processed by solution heat treatment. Trans. Nonferrous Met. Soc. China.

[34] Deng Y.L., Wan L., Wu L.H., Zhang Y.-Y., Zhang X.-M. (2011). Microstructural evolution of Al–Zn–Mg–Cu alloy during homogenization. J. Mater. Sci..

[35] Simsek I., Simsek D., Ozyurek D., Tekeli S. (2019). The Effect of the Aging Time on Microstructure and Mechanical Properties of the AA7075 Alloy after T6 Heat Treatment. Metallofiz. Noveishie Tekhnol..

[36] Shojaei K., Sajadifar S., Yapici G. (2016). On the mechanical behavior of cold deformed aluminum 7075 alloy at elevated temperatures. Mater. Sci. Eng. A.

[37] Maire E., Zhou S., Adrien J., Dimichiel M. (2011). Damage quantification in aluminium alloys using in situ tensile tests in X-ray tomography. Eng. Fract. Mech..

[38] Wu Y., Wang D.J., Liu Z., Liu G. (2019). A unified internal state variable material model for Ti 2AlNb-alloy and its applications in hot gas forming. Int. J. Mech. Sci..

[39] Yang L., Wang B., Liu G., Zhao H., Xiao W. (2015). Behavior and modeling of flow softening and ductile damage evolution in hot forming of TA15 alloy sheets. Mater. Des..

[40] Xu Z., Peng L., Jain M.K., Anderson D., Carsley J. (2021). Local and global tensile deformation behavior of AA7075 sheet material at 673oK and different strain rates. Int. J. Mech. Sci..

[41] Kowalewski Z.L., Hayhurst D.R., Dyson B.F. (1994). Mechanisms-based creep constitutive equations for an aluminium alloy. J. Strain Anal. Eng. Des..

[42] Murchú C., Leen S., O’donoghue P., Barrett R. (2017). A physically-based creep damage model for effects of different precipitate types. Mater. Sci. Eng. A.

[43] Pedersen K.O., Westermann I., Furu T., Børvik T., Hopperstad O.S. (2015). Influence of microstructure on work-hardening and ductile fracture of aluminium alloys. Mater. Des..

[44] Tham Y., Fu M., Hng H., Yong M., Lim K. (2007). Bulk nanostructured processing of aluminum alloy. J. Mater. Process. Technol..

[45] Masaki T., Masayuki K., Tatsuo M. (2007). Accelerated coarsening of MX carbonitrides in 12%Cr steels during creep deformation. ISIJ Int..

[46] Dyson B. (2000). Use of CDM in Materials Modeling and Component Creep Life Prediction. J. Press. Vessel. Technol..

[47] Perrin I.J., Hayhurst D.R. (1996). Creep constitutive equations for a 0.5Cr–0.5Mo–0.25V ferritic steel in the temperature range 600–675 °C. J. Strain Anal. Eng. Des..

[48] Sandström R., Lagneborg R. (1975). A model for hot working occurring by recrystallization. Acta Met..

[49] Liu G., Wang K., He B., Huang M., Yuan S. (2015). Mechanism of saturated flow stress during hot tensile deformation of a TA15 Ti alloy. Mater. Des..

[50] Tang X.-F., Wang B.-Y., Zhang N., Huo Y.-M., Zhou J. (2015). Modeling of microstructural evolution and flow behavior of superalloy IN718 using physically based internal state variables. Rare Met..

[51] Lin J., Dean T. (2005). Modelling of microstructure evolution in hot forming using unified constitutive equations. J. Mater. Process. Technol..

[52] Zhou J., Mu Y., Wang B. (2017). A damage-coupled unified viscoplastic constitutive model for prediction of forming limits of 22MnB5 at high temperatures. Int. J. Mech. Sci..

[53] Cao J., Lin J. (2008). A study on formulation of objective functions for determining material models. Int. J. Mech. Sci..

[54] Bai Q., Mohamed M., Shi Z., Lin J., Dean T. (2016). Application of a continuum damage mechanics (CDM)-based model for predicting formability of warm formed aluminium alloy. Int. J. Adv. Manuf. Technol..

[55] Bai Y., Teng X., Wierzbicki T. (2009). On the Application of Stress Triaxiality Formula for Plane Strain Fracture Testing. J. Eng. Mater. Technol..

